# Proteomics analysis of soluble secreted proteins of *Lutzomyia longipalpis* LL5 cells transfected with a dsRNA viral mimic: insights into cellular defense and repair signals

**DOI:** 10.3389/fcimb.2025.1638505

**Published:** 2025-09-11

**Authors:** Andrea Martins da Silva, Ilya Violeta Llanos Salamanca, Michel Batista, Fabricio Klerynton Marchini, Antonio Jorge Tempone, Erich Loza Telleria, Yara Maria Traub-Csekö

**Affiliations:** ^1^ Laboratório de Biologia Molecular de Parasitas e Vetores, Instituto Oswaldo Cruz – Fiocruz, Rio de Janeiro, Brazil; ^2^ Laboratório de Genômica Funcional, Instituto Carlos Chagas – Fiocruz, Curitiba, Brazil; ^3^ Plataforma Espectrometria de Massas – RPT02H, Instituto Carlos Chagas – Fiocruz, Curitiba, Brazil; ^4^ Department of Parasitology, Faculty of Science, Charles University, Prague, Czechia

**Keywords:** sand fly cell line, poly I:C, non-specific antiviral response, RNA degradation, cell repair, secreted proteins

## Abstract

Sand flies, which transmit diseases like leishmaniases, bartonellosis, and certain viruses, pose a significant public health threat. Our research focuses on the immune responses of *Lutzomyia longipalpis*, the primary vector for visceral leishmaniasis in the Americas. We use *L. longipalpis* LL5 cells as a model to study how sand flies respond to pathogens. These cells exhibit robust immune reactions, producing molecules mainly regulated by the Toll, IMD, Jak-STAT, and RNAi pathways. In previous studies, we detected a non-specific antiviral response in LL5 cells following double-stranded RNAs (dsRNAs) transfection. A previous complete secretome of these cells showed molecules resembling an interferon-like antiviral response when transfected with polyinosinic–polycytidylic acid (poly I:C), a synthetic dsRNA analog. In the current study, we analyzed soluble proteins secreted by LL5 cells after poly I:C transfection. Using comparative mass spectrometry, we examined protein composition of conditioned media depleted of exosomes at 24 h and 48 h. Most proteins uniquely expressed in the transfected groups had low abundance compared to the overall expressed proteins. Interactome prediction analysis revealed that at 24 h, the proteins uniquely found in the secretome of the transfected group were involved in RNA degradation and purine metabolism, while at 48 h they were linked to ribosomal proteins and signaling pathways such as Hedgehog, Transforming Growth Factor-beta (TGF-β), and Wingless/integrated (Wnt). We highlight increased abundance of the TGF-β-induced protein ig-h3 (24 h and 48 h), a Toll-like receptor 3 (48 h), and a hemocytin (48 h) in the secretion of transfected groups compared to the controls. We also performed an interaction analysis of proteins more secreted by the treated group at 24 h and 48 h. Unlike the interactome of uniquely identified proteins, few interactions were observed at 24 h, with a predominance of extracellular matrix and cell adhesion proteins. The set of proteins more secreted at 48 h presented more interactions than at 24 h, with emphasis on catabolic processes, including RNA degradation. These findings indicate that poly I:C transfection in LL5 cells induces the secretion of proteins involved in cellular defense and repair, revealing molecules involved in the LL5 non-specific antiviral response.

## Introduction

Sand flies can transmit parasites, bacteria, and viruses that cause diseases that affect humans and animals (Maroli et al., 2013; Ready, 2013). Phlebotomine species from the *Phlebotomus* and *Lutzomyia* genera are vectors of *Leishmania* parasites, important to public health in the Old and New World, respectively. In the Old World, sand flies are known vectors for Phlebovirus, Vesiculovirus, and Orbivirus, among others (Maroli et al., 2013; Jancarova et al., 2023). While definitive evidence of the New World sand flies transmitting viruses to humans is lacking, studies have detected various viruses in *Lutzomyia* species, as Phlebovirus (Palacios et al., 2011a, 2011b, 2015; De Carvalho et al., 2018; Hughes et al., 2019), Vesiculoviruses (Galindo et al., 1966; Travassos Da Rosa et al., 1984; Tesh et al., 1987; Corn et al., 1990). Other viruses in the Rhabdoviridae family (Aitken et al., 1975; Corn et al., 1990; Comer et al., 1992; Alkan et al., 2015), Peribunyaviridae (Shelokov and Peralta, 1967; Tesh et al., 1974; Aitken et al., 1975; Bonifay et al., 2023), Togaviridae (Bonifay et al., 2023), and Flaviviridae (Vasilakis et al., 2013) have also been identified in these vectors in the Americas.

The *Lutzomyia longipalpis* LL5 embryonic cell line (Tesh and Modi, 1983) is a useful study model that exhibits elaborate immune responses when exposed to various microbial challenges. For example, genes related to Toll, IMD, and Jak-STAT pathway regulators, as well as antimicrobial peptides, are differentially expressed after microbial challenges such as bacteria, yeast, and *Leishmania* parasites (Tinoco-Nunes et al., 2016; Telleria et al., 2021). These cells also present a non-specific antiviral response. For instance, after the transfection of several double-stranded RNA (dsRNA), LL5 cells suppress the luciferase reporter expression of a West Nile Virus-like particle. This response occurs regardless of the transfected dsRNA nucleotide sequence (Pitaluga et al., 2008). Such nonspecific antiviral response is reported in a few invertebrates, such as bees and shrimp (Flenniken and Andino, 2013; Wang et al., 2015), indicating a noncanonical regulatory pathway reminiscent of the interferon response in mammals, which is still not completely understood in arthropods.

To better understand the mechanisms behind the observed non-specific antiviral responses of LL5 cells, we previously transfected these cells with polyinosinic-polycytidylic acid (poly I:C) (Chamberlin and Patterson, 1965), a synthetic analog of double-stranded RNA (dsRNA) that mimics viral infection, and we analyzed the proteins present in the whole LL5 secretome released in the conditioned medium (Martins-da-Silva et al., 2018). Activation of the cellular immune response can occur when naïve cells come in contact with viral components or other signaling molecules from nearby infected cells, thus altering the repertoire of secreted proteins (Assil et al., 2015; Kalluri and LeBleu, 2020; Gurung et al., 2021). Such proteins can act as key players in cell signaling. In this previous study (Martins-da-Silva et al., 2018), the most abundant protein secreted by the poly I:C transfected LL5 cells is a phospholipid scramblase, a membrane protein involved in plasma membrane maintenance (Dal Col et al., 2022) with an additional role as an interferon-inducible protein that mediates antiviral activity (Luo et al., 2018). The other abundant protein is a forskolin-binding protein (FKBP), a member of the immunophilin family that participates in various biochemical processes such as protein folding, receptor signaling, protein trafficking, and transcription (Ghartey-Kwansah et al., 2018). These results from Martins-da-Silva et al. (2018) suggest that LL5 cells can present a nonspecific antiviral-like response similar to an interferon response in mammals.

Insects employ several antiviral responses. For example, one key mechanism is RNA interference (RNAi), where small interfering RNAs (siRNAs) guide the RNA-induced silencing complex (RISC) to degrade viral RNA (Zhu and Palli, 2020). In sand fly cell lines, the RNAi pathway is active (Tinoco-Nunes et al., 2016; Telleria et al., 2021; Alexander et al., 2023). Additionally, RNA decay pathways target viral RNA for degradation. Adenosine deaminase acting on RNA (ADAR) can deaminate adenosine residues in viral RNA, altering or degrading it (Samuel, 2012). Protein kinase R (PKR) detects double-stranded RNA and phosphorylates cellular and viral proteins, leading to the degradation of the viral RNA (Marques and Imler, 2016). Endoribonucleases, such as RNases, also cleave viral RNA, contributing to the antiviral cellular response through RNA degradation (Kingsolver et al., 2013; Cooper et al., 2014). These are molecular events directly involved in an antiviral cellular response.

Other immunity pathways also help eliminate viral infections. The Toll pathway’s pattern recognition receptors (PRRs) detect viral components, triggering a cascade that produces antimicrobial peptides and immune responses (Kingsolver et al., 2013). Regulatory pathways like TGF-β, which is important for tissue repair and controlling excessive immune reactions (Ishimaru et al., 2016), and the Wnt pathway, which maintains epithelial integrity (Ljungberg et al., 2019) and indirectly regulates antiviral gene expression, also contribute to viral resistance (Zhu and Zhang, 2013; Brutscher et al., 2017). These pathways highlight the complexity of the antiviral response within host cells.

Our current study presents the mass spectrometry analysis of the poly I:C transfected LL5 supernatant medium depleted of extracellular vesicles. This soluble fraction contains the proteins released from the cells into the extracellular environment that can play crucial roles in cell-cell communication, tissue development, immune response, and other physiological processes.

## Materials and methods

### LL5 cell culture

*L. longipalpis* embryonic LL5 cells (Tesh and Modi, 1983) were cultured at 30°C in L-15 medium (Sigma, Saint Louis, MO, USA) supplemented with 10% fetal bovine serum (Hyclone, Chicago, IL, USA), 10% Tryptose Phosphate Broth, and 1% antibiotics (penicillin 100 U/mL and streptomycin 100 mg/mL, Sigma).

### Transfection and collection of supernatants of conditioned medium

The experimental transfection mix consisted of 4 ng/mL of Lipofectin Transfection Reagent (Invitrogen, Carlsbad, CA, USA) mixed into L-15 medium (Sigma) containing 20% tryptose phosphate broth (Sigma) and 2 ng/mL of poly I:C (Chamberlin and Patterson, 1965), a synthetic analog of double-stranded RNA (Invitrogen). The transfection control used the same amount of Lipofectin Transfection Reagent mixed with the L-15 medium supplemented as described above, without the poly I:C. These two mixtures were added separately to 8 x 10^7^ LL5 cells distributed in 6-well flat bottom plates. After 24 h of incubation, the supernatant was carefully removed without disturbing the adherent cells, separated for subsequent centrifugation, and a fresh medium was added for an additional 24 h incubation. The supernatant was once again collected. The viability of the cells was confirmed through trypan blue staining, with over 98% of cells being viable in all experiments.

The supernatants were supplemented with Protease Inhibitor Cocktail 1X (Sigma) and centrifuged at 2000 x g for 10 min to pellet dead cells and large debris. The supernatants were subjected to another round of centrifugation at 10,000 x g for 30 min to eliminate any remaining cell debris and microvesicles. An additional centrifugation at 100,000 x g for 1 h was done to remove exosomes. The proteins in the supernatant were precipitated using trichloroacetic acid (TCA), as described below.

### Trichloroacetic acid precipitation

For protein precipitation, the supernatant was mixed with TCA 100% (Sigma) in the ratio of one volume of TCA to four volumes of conditioned medium. The mixture was then incubated at -20°C for 20 min. Subsequently, the samples were centrifuged at 10,000 x g for 10 min. The supernatant was carefully discarded, and the protein pellet was washed with cold acetone. After vortexing, the samples were centrifuged at 10,000 x g for 5 min. The resulting material was prepared for mass spectrometry analysis.

### Mass spectrometry analysis

Each experimental condition (transfected and control) was processed in duplicate for proteomics, for both 24 h and 48 h time points (n = 2 per group per time). The precipitated proteins were mixed with a solution containing 6 M urea, 2 M thiourea, and 10 mM Hepes. The proteins were reduced using 1 mM dithiothreitol (DTT) and 50 mM ammonium bicarbonate (ABC), followed by alkylation with 5.5 mM iodacetamide and 50 mM ABC. Before trypsinization, the samples underwent purification using detergent removal spin columns (Thermo Scientific, Rockford, IL, USA). Subsequently, the samples were digested in a solution of 50 mM ABC with trypsin (Promega, Madison, WI, USA) at a 1:50 trypsin-to-protein mass ratio and incubated at 24°C for 18 h. After trypsinization, trifluoroacetic acid (TFA) was added to achieve a final concentration of 0.5%. The peptides were desalted using homemade C18 spin columns. The peptide analysis was performed in triplicate using an LC-MS/MS system in a Thermo Scientific Easy-nLC 1000 coupled to an LTQ Orbitrap XL ETD at the mass spectrometry facility RPT02H/Carlos Chagas Institute - Fiocruz Paraná. Peptide separation occurred in a 15 cm fused silica column (inner diameter: 75 µm) packed in-house with reversed-phase ReproSil-Pur C18-AQ 3 µm resin from Dr. Maisch GmbH, Ammerbuch-Entringen. Chromatography runs were carried out with a flow rate of 250 nL/min, using a 120-min gradient from 5 to 40% MeCN in 0.1% formic acid. Peptide ionization was achieved by applying a voltage of 2.3 kV. The mass spectrometer operated in a data-dependent acquisition mode. Full-scan MS spectra were acquired in the Orbitrap analyzer within the range of 300 to 1,650 m/z, with a resolution of 60,000 at m/z 400, after accumulating to a target value of 500,000 in the C-trap. The ten most intense ions were sequentially isolated and fragmented in the linear ion trap using collision-induced dissociation with a target value of 30,000. The “lock mass” option was enabled at 445.120025 m/z in all full scans to improve the mass accuracy of precursor ions (Olsen et al., 2005). Protein identification was performed using the MaxQuant algorithm (version 1.4.1.2) (Cox and Mann, 2008; Cox et al., 2011), with default parameters unless otherwise specified. The search was conducted against a protein sequence database for *L. longipalpis*, which included 10,110 protein sequences from the VectorBase protein database (Amos et al., 2022) (downloaded on December 09, 2013), along with common contaminants and their respective reverse sequences to estimate the false discovery rate (FDR). Carbamidomethylation of cysteine was set as a fixed modification, while methionine oxidation and N-terminal acetylation (protein) were allowed as variable modifications. A threshold FDR of 0.01 was applied at both peptide and protein levels. Protein quantification was performed using a label-free approach, where the peptide peaks were detected as three-dimensional features (retention time versus signal intensity versus mass/charge) and aligned across the runs for comparison, as previously described (Luber et al., 2010). Following the identification and quantification by MaxQuant, zero values that represented the limit of detection were replaced with the minimum value found, which was 35,000, in order to obtain fold-changes for all comparisons. To obtain a representative value for each sample, the replicates were grouped using the median, which is less affected by extremely high or low values and provides a more typical value.

### *In silico* analyses

The amino acid sequences of the identified secreted proteins underwent bioinformatic analyses. To identify conserved protein domains and assign putative functions, we analyzed all amino acid sequences using profile hidden Markov models (HMM) via HMMER hmmscan (Potter et al., 2018) to query the protein sequences against the Pfam protein families database (Finn et al., 2016) with default parameters. For each protein, we retained the highest-confidence domain match, defined as the hit with the lowest E-value among all alignments. Hits with an E-value < 1e−5 were considered statistically significant and included in downstream analyses. We retrieved the protein log fold change expression values of the soluble fraction (current study) and the complete secretome (Martins-da-Silva et al., 2018) of the conditioned medium of LL5 transfected with poly I:C. The differential expression, as originally described in the corresponding methods of each study, was calculated compared to the mock-transfected LL5 control group (t-test, p < 0.05).

The Cytoscape software version 3.10.3 (Shannon et al., 2003) was used to create a network representation of the expression data from the soluble fraction (soluble proteins) or the complete secretome). We used the VectorBase accession numbers as nodes and the log fold change expression values for a color gradient representing upregulated (red) and downregulated (blue) proteins. The network edges indicate the corresponding soluble or exosome fractions of the transfected LL5 conditioned medium.

Domain descriptions were used to assist in biological interpretation and pathway enrichment.

Kyoto Encyclopedia of Genes and Genomes (KEGG) (Kanehisa et al., 2021) pathway enrichment was performed using the STRING database (Szklarczyk et al., 2023), using query protein names with *Drosophila melanogaster* as the reference organism due to its well-annotated genome. Results significance was assessed using FDR-adjusted p-values < 1e−5.

The protein signal peptide (SP) of secreted proteins was determined using the software tool Prediction of Signal Peptide “PrediSi” (Hiller et al., 2004), with the default settings.

The Search Tool for the Retrieval of Interacting Genes/Proteins (STRING) database version 12.0 was used for protein-protein interaction analysis (Szklarczyk et al., 2023).

## Results

**Figure 1:**
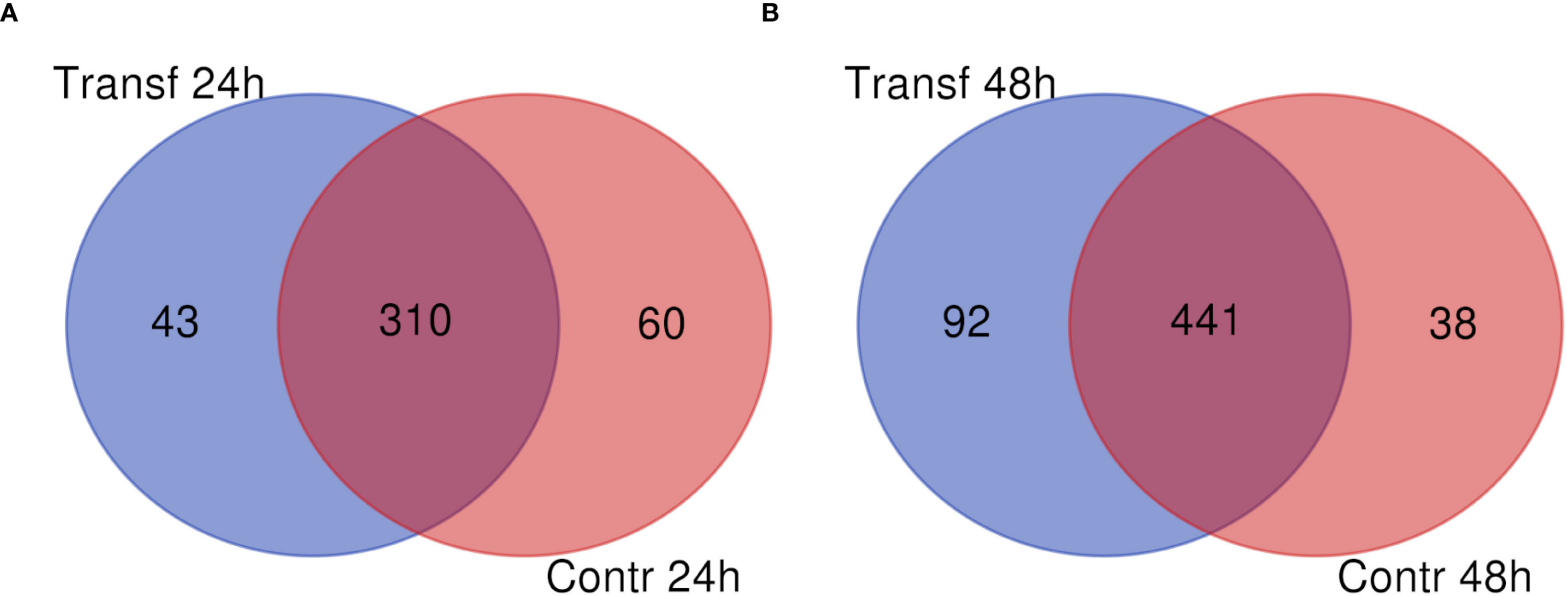
Number of soluble proteins identified in LL5 cells conditioned medium collected after poly I:C transfection. **(A)** medium collected at 24 h. **(B)** medium collected at 48 h post-transfection. Blue circles indicate the poly I:C transfected groups. Red circles indicate control groups.

To identify the proteins secreted by the *L. longipalpis* LL5 cells after the mimicked virus-like transfection, we conducted mass spectrometry analyses of the soluble proteins present in the conditioned medium collected at two different time points (24 h and 48 h) after poly I:C transfection compared to a control group collected at the same corresponding time points. A total of 611 proteins were identified, as indicated in **Supplementary Table 1**, from two biological replicates per condition. Most of these proteins (75%) were present throughout all analyzed time points in experimental and control samples. At 24 h post-transfection, 43 proteins were exclusively present in the experimental group (Transf 24 h), 60 proteins were present exclusively in the control group (Contr 24 h), and 310 proteins were shared among both groups (**Figure 1A**). At 48 h post-transfection, 92 proteins were present in the experimental group (Transf 48 h), 38 proteins were present exclusively in the control group (Contr 48 h), and 441 proteins were shared among both groups (**Figure 1B**).

Among the 43 proteins exclusively present in the poly I:C transfected group at 24 h, 41 presented similarity to protein domains in Pfam database. Among them, one of the most abundant proteins contained a myosin domain (PF00063). Other less expressed proteins had domains of a component of the nuclear pore complex (PF04097), a vitellinogen (PF09172), effectors that stimulate actin polymerization (PF07159), thioredoxin (PF00085 and PF13848), helicase (PF00270), prolyl 4-hydroxylase (PF08336), ubiquitin carboxyl-terminal hydrolase (PF00443), glucosamine-6-phosphate isomerase (PF01182), proteasome stabilizer (PF13001), Sm proteins involved in pre-mRNA splicing (PF01423), and sarcoma homology 2 domain (PF00017) (**Supplementary Table 1**).

Among the 92 proteins exclusively present in the transfected group at 48 h, 81 presented similarity to the HMM database, and one unknown protein was highly abundant. Among the less expressed proteins, there were proteins with domains belonging to an end-binding protein 1 (EB1) protein (PF03271), a ubiquitin-associated protein 2 (PF12478), another involved in U snRNA export from the nucleus (PF09088), a vacuolar protein involved in protein trafficking (PF03635), ribosomal protein S19 (PF00203) and L34e (PF01199), a 50S ribosome-binding GTPase (PF01926), a component of the nuclear pore complex (PF04097) (also present at 24 h), a metalloprotease M16C (PF08367), pre RNA processing ribonucleoproteins (PF01798), caprin-1 protein involved in cellular proliferation, innate immune response and synaptic plasticity (PF18293), a nuclear protein localization protein 4 (NPL4) associated to nuclear transport and protein degradation (PF05021), a minichromosome maintenance (MCM) involved in the initiation of eukaryotic DNA replication (PF00493), alpha/beta hydrolases (PF02230), a pheromone binding protein (PF01395), a glucose-6-phosphate dehydrogenase (PF02781), and an importin subunit alpha-2 involved in nucleocytoplasmic transport (PF16186) (**Supplementary Table 1**).

**Figure 2:**
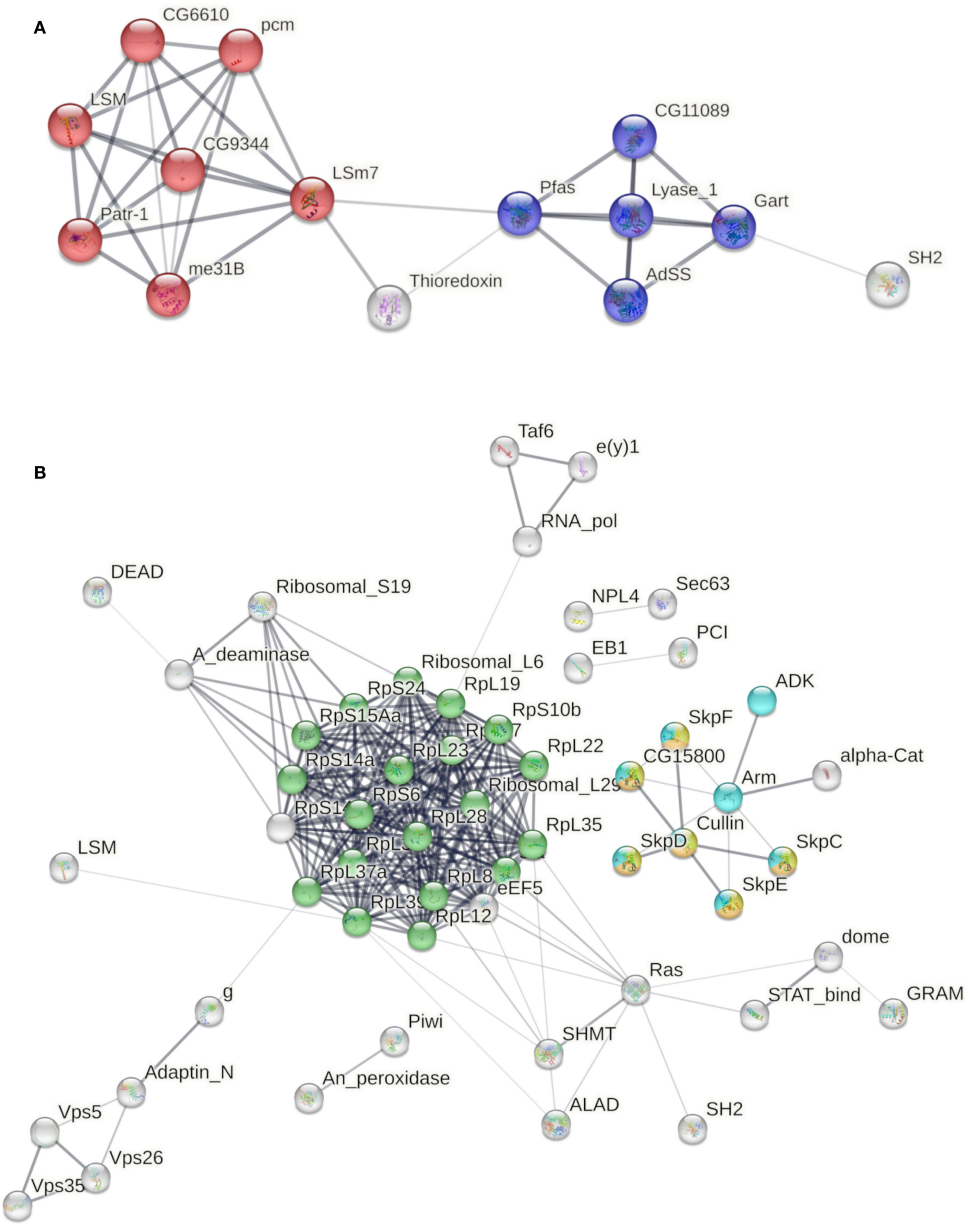
Interactome of proteins uniquely secreted by poly I:C transfected LL5 cells. **(A)** Functional enrichment corresponding to KEGG pathways from transfected cells at 24 h. Red color indicates RNA degradation, and blue color indicates purine metabolism pathways. **(B)** Functional enrichment corresponding to KEGG pathways from transfected cells at 48 h. Green color indicates the ribosome pathway. Yellow, orange, and light blue colors indicate the Hedgehog, TGF-β, and Wnt signaling pathways, respectively. Line thickness indicates the strength of data support. The edges indicate both functional and physical protein associations (FDR-adjusted p-values < 1e−5).

We analyzed the interactome of the proteins uniquely present in the poly I:C transfected group at 24 h. Among them, 17 matched with *D. melanogaster* proteins available on the String interactome database (**Supplementary Table 2**). We found the most significant functional enrichment corresponding to KEGG pathways on RNA degradation (false discovery rate 3.3e-09) and purine metabolism (false discovery rate 8.1e-05) (**Figure 2A**). At 48 h, 39 uniquely identified proteins in the transfected group matched with the *D. melanogaster* String database. The most significant functional enrichment corresponding to KEGG pathways were ribosome (false discovery rate 5.09e-20), Hedgehog, TGF-β, and Wnt signaling pathways (false discovery rate 4.07e-06) (**Figure 2B**).

**Figure 3:**
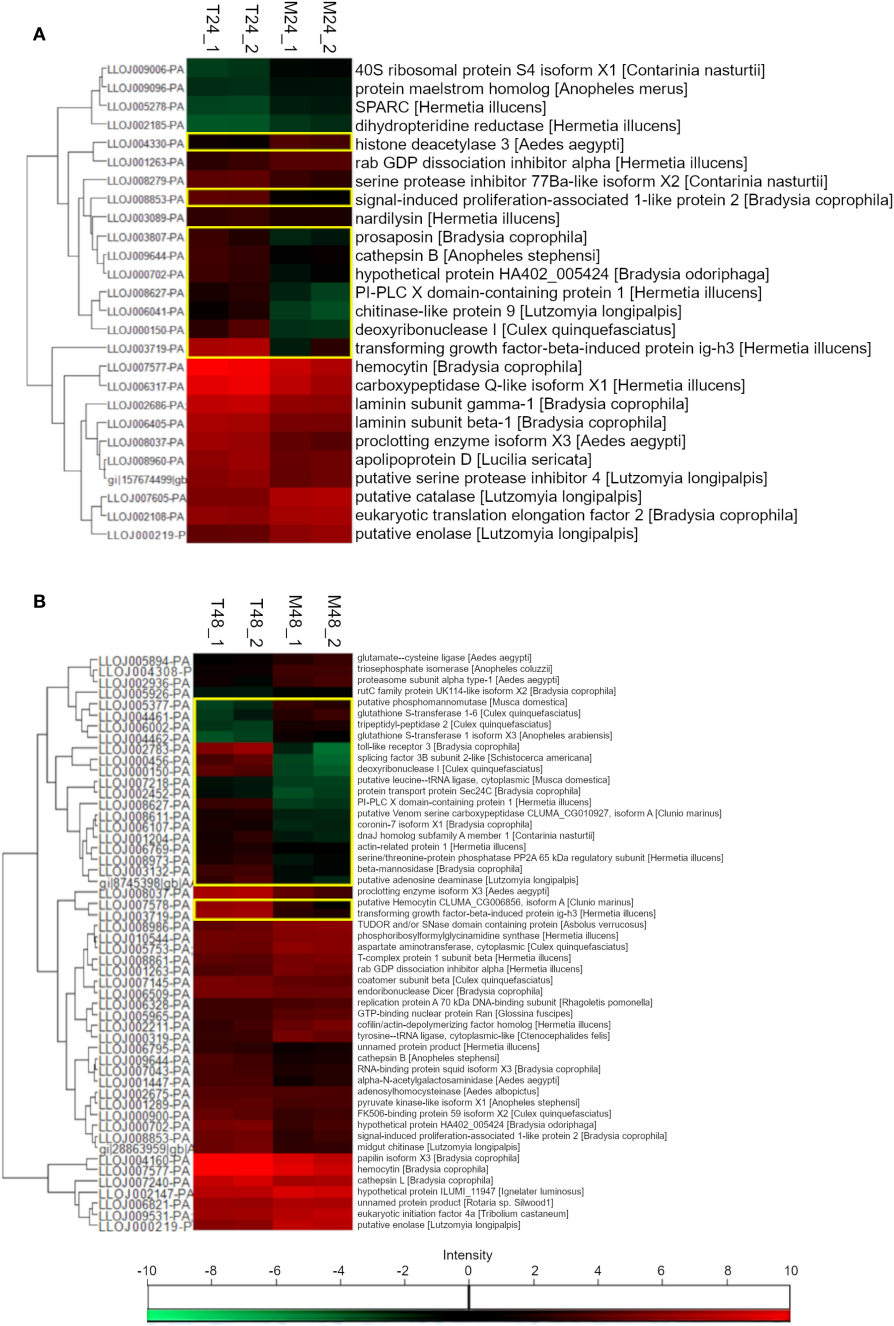
Heatmap representation of protein abundance. Secreted proteins 24 h **(A)** and 48 h **(B)** in transfected (T24_1, T24_2, T48_1, and T48_2) and control (M24_1, M24_2, M48_1, and M48_2) LL5 cells. Red color indicates increased detection. Green color indicates decreased detection. Protein sequences are identified by the VectorBase ID on the left and the protein description of the best match against the NCBI database on the right side of the image. Yellow rectangles indicate differentially secreted proteins (t-test, p<0.05). The lower inset represents the color scale for protein intensity.

Because many proteins were present in both experimental and control groups, we analyzed whether there were significant upregulated or downregulated protein secretion based on statistical testing among these two groups. We analyzed those proteins identified in both transfected and control groups at 24 h (310 proteins) and 48 h (441 proteins) post-transfection out of the 611 total proteins. Among the proteins with label-free quantification (LFQ) intensity in all replicates, we identified 26 proteins at 24 h, and 9 of them were differentially secreted in transfected compared to the control group (**Figure 3A**). One protein had the highest similarity with histone deacetylase 3 from the mosquito *Aedes aegypti* and was increased in the control group. Eight other proteins were increased in the transfected group and had the highest similarity with prosaposin, signal-induced proliferation-associated 1-like protein 2, and a hypothetical protein from *Bradysia* fungi; phosphatidylinositol-specific phospholipase C, X (PI-PLC X) domain-containing protein 1 and a TGF-β-induced protein ig-h3 from the black soldier fly *Hermetia illucens*; cathepsin B from *Anopheles stephensi*; deoxyribonuclease I from *Culex quinquefasciatus*; and a chitinase-like protein 9 from *L. longipalpis*. Among the proteins with LFQ intensity in all replicates, at 48 h, we identified 52 proteins, and 19 of them were differentially secreted in transfected compared to the control group (**Figure 3B**). Four of them were decreased in the transfected group: one similar to a predicted phosphomannomutase from the house fly *Musca domestica* two similar to mosquito’s glutathione S-transferases, and another similar to a tripeptidyl-peptidase 2 from *C. quinquefasciatus*. Fifteen proteins were increased in the transfected group with highest similarity with: toll-like receptor 3, transport protein Sec24C, coronin-7 isoform X1, and beta-mannosidase from the fungus *Bradysia coprophila*; splicing factor 3B subunit 2-like from the American grasshopper *Schistocerca americana*; a putative Venom serine carboxypeptidase from the non-biting midge *Clunio marinus*; chaperone DnaJ homolog subfamily A, also known as heat shock protein 40 kD (Hsp40), from the swede midge *Contarinia nasturtii*; actin-related protein 1, serine/threonine-protein phosphatase PP2A 65 kDa regulatory subunit isoform X2, PI-PLC X domain-containing protein 1 and TGF-β-induced protein ig-h3 from *H. illucens* (both increased at 24 h); a cytoplasmic leucine-tRNA ligase from *M. domestica*; glutathione S-transferase 1-6, tripeptidyl-peptidase 2, deoxyribonuclease I from *C. quinquefasciatus* (also increased at 24 h); glutathione S-transferase 1 isoform X3 from *Anopheles arabiensis*; and a putative adenosine deaminase from *L. longipalpis*.

**Figure 4:**
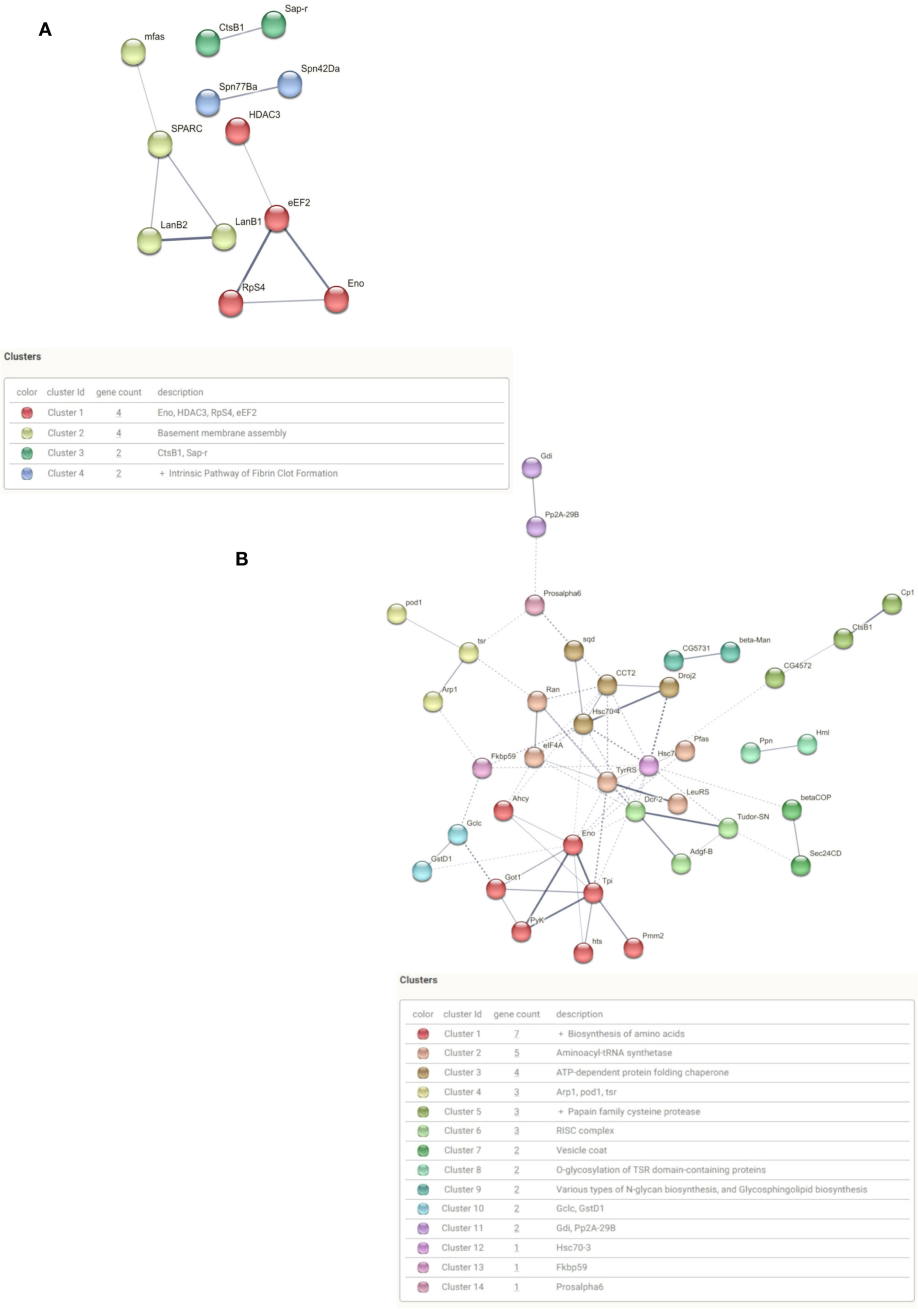
Interaction analysis of differentially secreted protein after poly I:C treatment. **(A)** Interaction of secreted protein clusters 24 h after poly I:C. **(B)** Interaction of secreted protein clusters 48 h after poly I:C.

We also performed an interaction cluster analysis using proteins that were most secreted at 24 h and those that were most present at 48 h. The protein groups presented PPI enrichment p-values of 3.18 x 10^-3^ and 2.24 x 10^-11^, respectively. These values indicate that the networks have significantly more interactions than expected. The group of 26 proteins prevalent at 24 h showed few interactions, with the formation of four clusters enriched in extracellular functions (**Figure 4A**). Gene ontology analysis of this group showed enrichment of biological processes related to basal membrane assembly (GO:0070831) and extracellular matrix organization (GO:0030198). The only enriched KEGG pathway was the ECM-receptor interaction (dme04512) (**Supplementary Data Sheet 1**). The 52 proteins analyzed at 48 h showed a greater number of interactions, with the formation of 14 clusters. (**Figure 4B**). Gene ontology analysis of the most secreted proteins 48 h after poly I:C treatment shows enrichment of catabolic biological processes (GO:0009056; GO:1901575; GO:1901136). This set of secreted proteins showed the highest enrichment of KEGG pathways related to amino acid biosynthesis (dme01230); cysteine and methionine metabolism (dme00270), and glycolysis/gluconeogenesis (dme00010) (**Supplementary Data Sheet 2**).

Among the clusters identified among the 52 most present proteins in 48 h, the RISC complex (cluster 6) stands out. This cluster is formed by the proteins Tudor staphylococcal nuclease (Tudor-SN), Adenosine deaminase-related growth factor B (Adgf-B) and Dicer-2 (**Figure 3B**). The Tudor-SN protein is an endonuclease with activity on DNA and RNA substrates. In *Drosophila*, it is involved in translation regulation, piwi regulation and transposons control in the germline through its association with the with the RISC complex. The Adgf-B protein has adenosine deaminase activity. It is in the inosine biosynthetic process and the adenosine catabolic process. Dicer-2 encodes a member of the RNase III family of double-stranded RNA-specific endonucleases. It acts in the RNAi pathway by cutting dsRNA into siRNAs and helps defend flies against viral infection, particularly RNA viruses. It also processes long, partially double-stranded endogenous transcripts (hairpin RNAs) into endo-siRNAs.

Since one of the mechanisms of protein secretion is through signal peptides, we investigated the presence of signal peptides in the set of proteins with LFQ intensity in all replicates (**Figures 3A, B**). Using the Phobius and PrediSi prediction tools, we identified 13 proteins secreted at 24 h, and 12 proteins at 48 h contained signal peptide (**Table 1**).

**Table 1:** Prediction of Signal Peptides (Phobius and PrediSi).

Transfected group	Phobius	PrediSi
24 h (26 proteins)	13 (50%)	13 (50%)
48 h (52 proteins)	12 (23.1%)	12 (23.1%)

## Discussion

Our study of *L. longipalpis* LL5 cells transfected with poly I:C mimics a dsRNA virus infection and provides insights into their antiviral-like response. Virus detection in *Lutzomyia* genus is geographically widespread, with reports from Brazil to USA (Fonseca et al., 2021; Jancarova et al., 2023; Tempone et al., 2024). While the frequency of infection varies depending on the specific virus and sand fly species, as well as the geographic location, many of the sand fly viruses appear to establish persistent, non-lethal infections in the sand fly (Mavale et al., 2006; Alkan et al., 2013; Laroche et al., 2024).

Although poly I:C is not derived from any specific viral genome and does not correspond to the nucleotide sequences of known viruses transmitted by sand flies, it is widely used as a synthetic analog of dsRNA—a molecular pattern recognized by host cells as a hallmark of viral infection (Wang et al., 2015). During the replication of many RNA viruses, including those from the families Reoviridae, Flaviviridae, Rhabdoviridae, and Phenuiviridae (which includes Phlebovirus), dsRNA molecules are produced as replication intermediates, typically in the form of double-stranded replicative forms or transient RNA duplexes formed during synthesis (Tao and Ye, 2010; Rampersad and Tennant, 2018). Poly I:C mimics the structural and molecular features of these viral dsRNA intermediates by forming long, stable duplexes. Nevertheless, this synthetic molecule does not replicate other hallmarks of a viral infection, such as productive viral replication, cytopathic effects, or virulence-associated mechanisms, including host cell lysis, manipulation of host gene expression by viral proteins, or subversion of immune signaling pathways (Alexopoulou et al., 2001; Matsumoto and Seya, 2008; Wang et al., 2015). Instead, poly I:C primarily mimics the presence of viral dsRNA, triggering cellular recognition and innate immune signaling without inducing virus-specific pathogenic outcomes.

Previous analyses of the LL5 complete secretome showed that these cells can mount a response mirroring vertebrate interferon response (Martins-da-Silva et al., 2018). Although invertebrates lack a canonical IFN system, nucleic acid stimulation and viral infections can activate an inducible non-specific antiviral response that shares several features with the vertebrate IFN system (Wang and He, 2019; Marques et al., 2024). By employing mass spectrometry to analyze the soluble proteins in the conditioned medium from two biological replicates at two distinct time points (24 h and 48 h post-transfection), the current study reveals a distinct profile of the proteins involved in the complex response to the mimicked viral infection.

Nearly 10% and 16% of the overall secreted proteins were identified uniquely at 24 h and 48 h post-transfection, respectively, suggesting that they are involved in the response to poly I:C transfection. Conversely, approximately 14% and 7% (24 h and 48 h, respectively) of the proteins were found only in the control group, indicating that these proteins may be involved in routine cellular activities or responses unrelated to viral mimicry. Additionally, 75% and 77% (24 h and 48 h, respectively) of the proteins were shared between both groups, reflecting that most of the secreted proteins belong to common activated functions or pathways, emphasizing that many cellular processes remain active despite the viral mimicry. The increased number of shared secreted proteins at 48 h indicates that the effect of poly I:C may be fading over time.

The overview of the conserved domains of the proteins uniquely detected in the transfected group at 24 h showed proteins involved in cellular structure, indicating that cytoskeletal dynamics may be altered in response to viral mimicry (Walsh and Naghavi, 2019; Khorramnejad et al., 2021). Other proteins uniquely identified in the same group included components associated with critical cellular functions such as nucleocytoplasmic transport, which is vital during viral infections when pathogens often hijack nucleic acid trafficking (Yarbrough et al., 2014; Shen et al., 2021); cytoskeletal rearrangement, potentially facilitating immune cell migration or phagocytosis in an *in vivo* model (Mostowy and Shenoy, 2015; Mylvaganam et al., 2021); antioxidant properties, indicating a response to oxidative stress (Peterhans, 1997; Espinosa-Diez et al., 2015); and proteins involved in RNA processing, highlighting the importance of mRNA splicing and processing in mounting an effective immune response (Lee et al., 2019; Cui et al., 2022).

At 48 h post-transfection, the conserved domain analysis of the proteins uniquely identified in the transfected group contained one unknown protein that was notably abundant, warranting further investigation to elucidate its function. Other interesting proteins in the same group are involved in microtubule dynamics that are crucial for intracellular transport and signaling during immune responses (Seo and Gammon, 2022), protein degradation pathways that are essential for regulating protein levels during stress responses (Flick and Kaiser, 2012; Rosche et al., 2021), ribosomal proteins suggesting the activation of protein synthesis as part of the cellular response to viral mimicry (Wang et al., 2022), probably as an attempt to translate the poly I:C sequence, and a protein linked to cellular proliferation and innate immune responses, highlighting its potential role in modulating cell growth during infection.

Through the String interactome, we can highlight the biological pathways that are significantly impacted during the response to the poly I:C. At 24 h post-transfection, the most significant functional enrichment was observed in two KEGG pathways. One of them is RNA degradation, a critical mechanism for controlling viral replication and preventing the accumulation of viral RNA within cells (Houseley and Tollervey, 2009; Singh et al., 2017). The activation of this pathway suggests that LL5 cells are actively engaging in antiviral strategies by degrading the potentially harmful RNA molecules. This is consistent with known roles of exonucleases, deadenylases, and decapping enzymes in innate immunity in insects and other eukaryotes (Cui et al., 2022). The other is purine metabolism, which reflects an increased demand for nucleotides during the immune response, particularly for synthesizing nucleic acids as part of cellular repair and proliferation processes (Dolezal et al., 2019; Ariav et al., 2021). This metabolic shift could provide the necessary building blocks for synthesizing new cellular components required during stress responses.

At 48 h post-transfection, the interactome analysis identified the ribosome pathway’s prominence, highlighting an upregulation in protein synthesis (Wang et al., 2022). The increased ribosomal activity suggests that LL5 cells are prioritizing the production of proteins potentially for defense mechanisms, signaling, and cellular repair. Other enriched signaling pathways indicate a complex regulatory network activated in response to viral mimicry. For instance, hedgehog signaling is known to play roles in cell differentiation and development (Villarreal et al., 2015) but may also influence immune responses by regulating cell fate decisions (Benson et al., 2004). The TGF-β signaling is involved in various cellular processes, including immune regulation and tissue repair (Ishimaru et al., 2016; Massagué and Sheppard, 2023), suggesting that LL5 cells may be engaging in mechanisms to restore homeostasis following viral challenge. In addition, the Wnt signaling contributes to cell proliferation and differentiation (Teo and Kahn, 2010), further emphasizing the dynamic nature of cellular responses during infection.

The findings of uniquely secreted proteins in the transfected group from both time points illustrate how LL5 cells adapt to engage specific pathways and resist the viral mimicry effectively. The activation of RNA degradation suggests an activation of RNA surveillance and purine metabolism pathways, reflecting increased nucleotide turnover at 24 h, which in turn reflects a metabolic reprogramming and an immediate antiviral response consistent with an early antiviral defense phase. The later activation of ribosomal biogenesis and key signaling pathways at 48 h reflects a transition toward recovery and adaptation. The secretion of such dynamic sets of proteins to the extracellular milieu can act as damage-associated molecular patterns (DAMPs), influencing neighboring cells, systemic immunity, or even facilitating tissue remodeling, analogous to innate immune signaling in other invertebrates (Su et al., 2024). It is possible that secreted metabolic and RNA-related proteins at 24 h served as both effectors and messengers in shaping later immune response at 48 h involving hedgehog, TGF-β, and Wnt signaling; however, this interpretation has not yet been tested. Cluster interaction analysis of the proteins differentially secreted by the treated group at both time points reveals that, similar to what was observed among the exclusively secreted proteins, the cellular response to dsRNA challenge leads to a bimodal response. Initially, we see an increase in proteins related to processes that structure the extracellular matrix environment. Later, we observe a shift among the most secreted proteins, now with an emphasis on catabolic processes, which also involve RNA degradation. This temporal analysis highlights the dynamic nature of immune responses, where initial defensive actions evolve into broader regulatory mechanisms to sustain cellular function under stress.

The comparison of proteins present in both transfected and control groups at 24 h and 48 h post-transfection revealed potential mechanisms of immune modulation. For instance, at 24 h, the protein similar to *A. aegypti* HDAC3 (Gaddelapati et al., 2022) had increased secretion in the control group; thus, by analogy, there was a proportional reduction in the transfected group. The increase of HDACs is often associated with responses to cellular stress, indicating that the cells from the control group are ready to mitigate the potential transfection challenges, whereas the transfected cells are less capable of using this molecular repertoire to maintain the chromatin structure and gene regulation (Somers et al., 2023). In contrast, the transfected group increased the secretion of eight other proteins. The prosaposin is involved in cell survival and differentiation (Leonova et al., 1996), indicating another protective mechanism activated against cellular stress. The TGF-β-induced protein ig-h3 is involved in tissue repair and immune modulation (Thapa et al., 2007), indicating an active response to the viral mimicry, and the chitinase-like protein 9 suggests a role in pathogen defense or tissue remodeling (Arakane and Muthukrishnan, 2010) with activity for soluble polymeric substrates as seen in *Drosophila* (Zhu et al., 2008). The presence of these proteins indicates that LL5 cells are initiating specific immune responses upon exposure to poly I:C, focusing on survival and repair mechanisms.

At 48 h, the analysis of differentially secreted proteins in both transfected and control groups revealed four proteins with decreased presence in the transfected group. The decrease of a tripeptidyl-peptidase 2 suggests alterations in proteolytic processes (Tomkinson and Lindås, 2005). In addition, a predicted phosphomannomutase from the house fly *M. domestica* (Ray and Heslop, 1963) and two glutathione S-transferases from mosquitoes (Ranson and Hemingway, 2005) showed that proteins typically involved in detoxification processes were reduced and suggested a reduced need for detoxification as cells adapt to viral mimicry. Conversely, fifteen proteins were increased in the transfected group, including the Toll-like receptor 3 (TLR3), which is pivotal in recognizing viral RNA and initiating immune responses (Leulier and Lemaitre, 2008; Perales-Linares and Navas-Martin, 2013), underscoring its role in the antiviral response; a heat shock protein 40 (Hsp40) known for its chaperone functions during stress conditions (King and Macrae, 2015; Zhang and Yu, 2022), indicating that LL5 cells are actively managing protein folding and preventing aggregation under stress; a coronin-7 isoform X1 involved in actin dynamics and cellular signaling pathways, suggesting enhanced cytoskeletal rearrangements necessary for immune responses (Shina et al., 2010; Yumura et al., 2022).

Interestingly, one protein was uniquely present in the transfected cells, and three others were increased in the transfected group at both time points: a component of the nuclear pore complex, phosphatidylinositol-specific phospholipase C, X (PI-PLC X) domain-containing protein 1, a TGF-β-induced protein ig-h3, and a deoxyribonuclease I. The presence of these proteins in transfected cells highlights how these cells are responding to viral mimicry. By enhancing nucleocytoplasmic transport, modulating lipid signaling pathways, promoting tissue repair, and managing extracellular DNA, these proteins collectively contribute to the cell’s ability to adapt and respond effectively to stressors associated with viral infections.

When we analyzed the proteins that are present in both groups and that were more secreted in the cells treated with poly I:C, we observed that in 24 h there is an increase in the secretion of proteins involved in the structuring of extracellular components, while in 48 h the most secreted proteins are more related to cellular catabolic processes.

In summary, the differential secretion of these proteins highlights the adaptive nature of LL5 cells when faced with viral mimicry. The initial response at 24 h focuses on survival and repair mechanisms, while by 48 h, there is a shift towards more robust immune signaling and stress management. The upregulation of TLR3 and Hsp40 secretion indicates an escalation of antiviral defenses as the cells continue to respond to poly I:C.

In other insect cell lines challenged by dsRNA viral-like molecules also showed a non-specific response. For example, the mosquito *C. quinquefasciatus* ovary-derived cells upregulated multiple Toll pathway receptors (Prince et al., 2023), while the *A. aegypti* Aag2 cells activated the IMD pathway (Russell et al., 2021) after poly I:C transfection. Earlier studies on lepidopteran cells showed that the silkworm *Bombyx mori* BmN4 and fall armyworm *Spodoptera frugiperda* Sf21 pose a nonspecific effect after poly I:C transfection (Sakashita et al., 2009). More interestingly, in the honey bee *Apis melifera*, the dsRNA challenge revealed an RNAi-independent non-specific antiviral immune mechanism (Flenniken and Andino, 2013).

The prediction of the signal peptides in the amino acid sequences showed that only a small fraction of the identified proteins, approximately 12% at 24 h and 4% at 48 h post-transfection, were secreted via canonical pathways involving the endoplasmic reticulum and Golgi apparatus (Mijaljica et al., 2006; Balmer and Faso, 2021). This indicates that the majority of the proteins were likely secreted through non-canonical mechanisms. Specifically, two major unconventional pathways are known to be involved in the secretion of soluble proteins synthesized in the cytoplasm. Type I secretion involves direct translocation across lipid pores in the plasma membrane. In type III secretion, cytoplasmic proteins are recruited into vesicular compartments of the endocytic membrane system that subsequently fuse with the plasma membrane to release proteins into the extracellular space (Dimou and Nickel, 2018).

Interestingly, the most abundant proteins secreted by the poly I:C transfected LL5 cells detected in the previous complete secretome (Martins-da-Silva et al., 2018), a phospholipid scramblase, with a role as an interferon-inducible protein that mediates antiviral activity and a forskolin-binding protein, a member of the immunophilin family, were not found in the present study. This is most probably explained by the presence of these proteins in exosomes, which were removed in the present study. The comparative analysis between the soluble and the complete secreted proteomes of LL5 cells transfected with poly I:C (see also **Supplementary Data Sheet 3**), where a predominance of proteins associated with exosomes was observed, reveals both overlapping and distinct features in the secretory response of these insect cells. Notably, although 48 proteins were identified as differentially regulated in both fractions across the 24 h and 48 h time points, their expression profiles often diverged depending on the secretory route. For instance, at 24 h post-transfection, proteins such as signal-induced proliferation-associated protein and prosaposin domain-containing protein were upregulated in the soluble fraction but not in the complete proteome, while other proteins like FKBP and GST_N were significantly modulated in the complete secretome only, suggesting differential sorting or retention mechanisms. By 48 h, although hemocytin and PMM showed concordant regulation across both compartments, eIF2A and GST_N displayed opposite or compartment-specific regulation (Basisty et al., 2020). These discrepancies underscore a dynamic and potentially compartmentalized cellular response to viral mimic stimulation.

These differences reflect the temporal and functional divergence of the secretory routes mobilized in response to viral mimicry. The soluble secretome may represent a more rapid, expansive means of extracellular signaling, incorporating metabolic enzymes, signaling ligands, and even components of translational machinery with potential immunomodulatory effects. In contrast, the exosomal pathway appears to orchestrate a more regulated and selective export of immune and stress-related proteins, possibly tuned for cell-to-cell delivery and long-range effects (Munoz-Perez et al., 2021). Together, the two secretomes illustrate distinct yet potentially complementary aspects of the innate antiviral landscape in sand fly cells.

Importantly, this study differs from Martins-da-Silva et al. (2018) not only in the focus on soluble secreted proteins but also in the cellular context captured. While exosomes represent a vesicle-based, potentially regulated route of intercellular communication, the soluble proteome likely reflects a range of secreted proteins, including freely diffusing mediators of local and systemic responses (Samuelson and Vidal-Puig, 2018). The soluble fraction may also contain proteins related to acute-phase responses, stress signaling, or passive leakage, not captured within the exosomal compartment. This distinction highlights the complementary nature of both datasets: the exosomal proteome reveals targeted export of regulatory components, while the soluble proteome captures the immediate extracellular milieu, including potential effector proteins (Basisty et al., 2020). Together, the two studies provide a more integrated view of how LL5 cells modulate their secretory machinery in response to dsRNA analogs and may contribute differentially to cell-cell signaling, immune modulation, and antiviral defense.

We emphasize that this is an exploratory *in vitro* study aimed at characterizing the soluble secreted protein response of LL5 sand fly cells following exposure to poly I:C. The experimental design is constrained by the limited yield of secreted proteins after the depletion of extracellular vesicles, which restricts downstream proteomic analyses. Accordingly, the interpretation of the data should be made with caution, particularly regarding broader physiological relevance. Future studies will be required to validate and expand these findings *in vivo*, including oral administration or microinjection of poly I:C into adult *Lutzomyia longipalpis* to evaluate systemic immune responses.

In conclusion, our results showed that the LL5 cells initially secreted molecules involved in RNA processing, cell repair, and maintenance in response to the dsRNA viral mimicry. Then, they switched to protein recycling and a more complex immune response. Concomitantly, there was a reduction in some stress and detoxification response mechanisms. Their identification provides a novel set of candidate markers for immune activation in sand flies, offering new targets for functional studies of immune priming antiviral immunity, systemic signaling, and host–virus–parasite interactions.

## Data Availability

The original contributions presented in the study are included in the article and **Supplementary Material**. Further inquiries can be directed to the corresponding author.
