# Supplementary material for: Proteomics analysis of soluble secreted proteins of Lutzomyia longipalpis LL5 cells transfected with a dsRNA viral mimic: insights into cellular defense and repair signals
Source: Front Cell Infect Microbiol. 2025 Sep 11;15:1638505. doi: 10.3389/fcimb.2025.1638505 (PMC12460361; doi:10.3389/fcimb.2025.1638505)

Gene Ontology and KEGG pathway enrichment of differentially expressed proteins at 24 h post poly I:C transfection

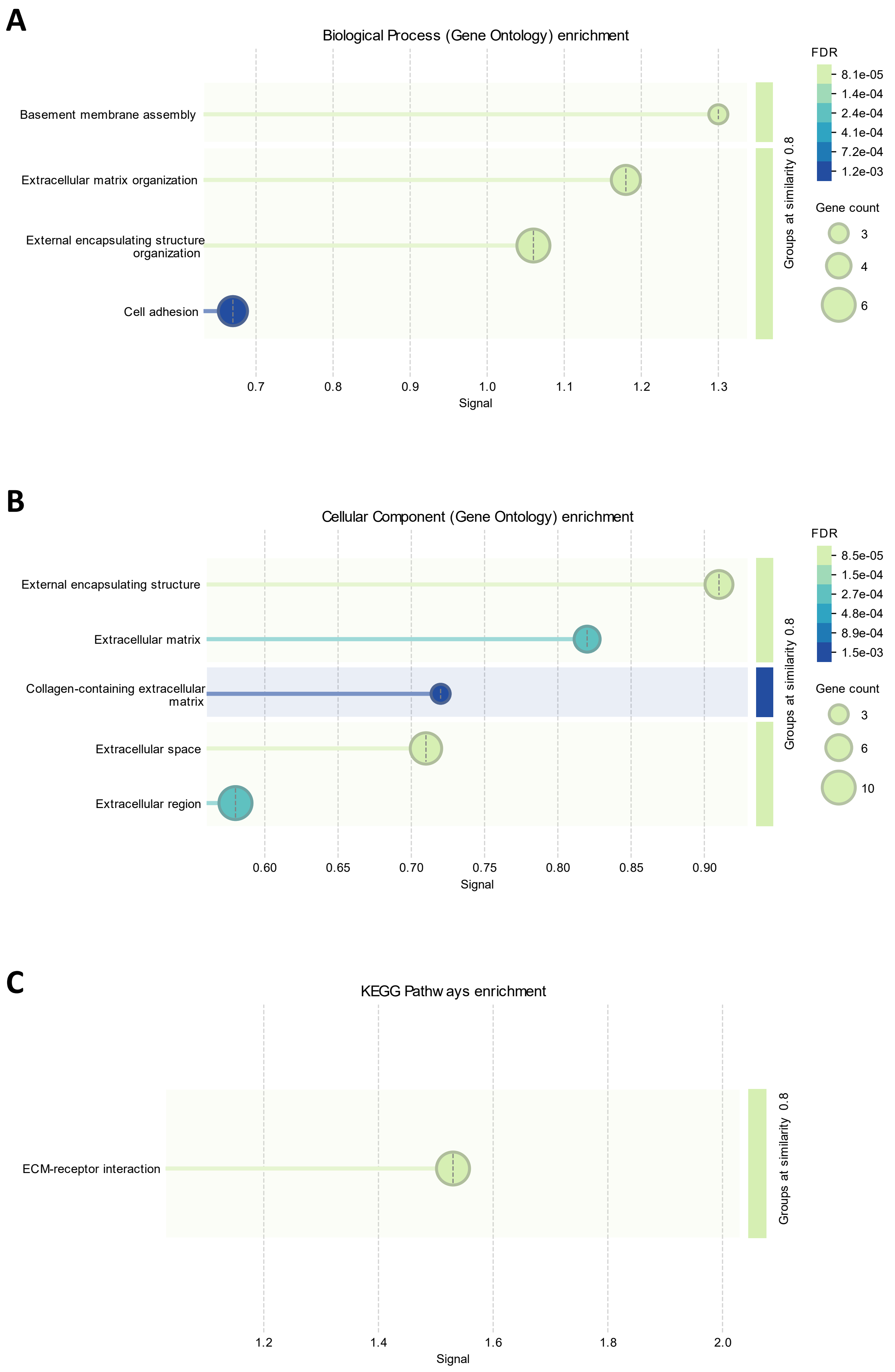

Supplement: Supplementary file 1 [file DataSheet1.pdf]
