# Supplementary material for: Proteomics analysis of soluble secreted proteins of Lutzomyia longipalpis LL5 cells transfected with a dsRNA viral mimic: insights into cellular defense and repair signals
Source: Front Cell Infect Microbiol. 2025 Sep 11;15:1638505. doi: 10.3389/fcimb.2025.1638505 (PMC12460361; doi:10.3389/fcimb.2025.1638505)
Supplement: Supplementary file 3 [file DataSheet3.pdf]

**Analysis of proteins differentially expressed by LL5 cells transfected with poly I:C identified in the soluble fraction (current study) or complete secretome (Martins-da-Silva et al. 2018).**

## Methods

We retrieved the protein log fold change expression values (Supplementary Table 3) of the soluble fraction (current study) and the complete secretome (Martins-da-Silva et al. 2018) of the conditioned medium of LL5 transfected with poly I:C. The differential expression, as originally described in the corresponding methods of each study, was calculated compared to the mock-transfected LL5 control group (t-test, $p<0.05$).

The Cytoscape software version 3.10.3 (Shannon et al. 2003) was used to create a network representation of the expression data from the soluble fraction (soluble proteins) or the complete secretome). We used the VectorBase accession numbers as nodes and the log fold change expression values for a color gradient representing upregulated (red) and downregulated (blue) proteins. The network edges indicate the corresponding soluble or exosome fractions of the transfected LL5 conditioned medium.

## Results

Among the proteins that had significant differential expression (t-test, $p<0.05$), we identified 48 proteins that were present in both soluble and complete secretomes, either at 24 h or (Martins-da-Silva et al.

2018)

| VectorBase ID | Protein Name | Fold change at 24h | VectorBase ID | Protein Name | Fold change at 24h |
|---|---|---|---|---|---|
| LLOJ003719 | Fasciclin | 4.49 | LLOJ008853 | Rap_GAP | 2.43 |
| LLOJ002783 | unknow | 3.43 | LLOJ006041 | Glyco_hydro_18 | 2.37 |
| LLOJ000150 | unknow | 3.09 | LLOJ008627 | unknow | 2.20 |
| LLOJ008611 | Peptidase_S10 | 2.91 | LLOJ003807 | SapA | 2.05 |
| LLOJ007210 | Ceramidase_alk | 2.60 | AAF78901 | A_deaminase | 2.04 |

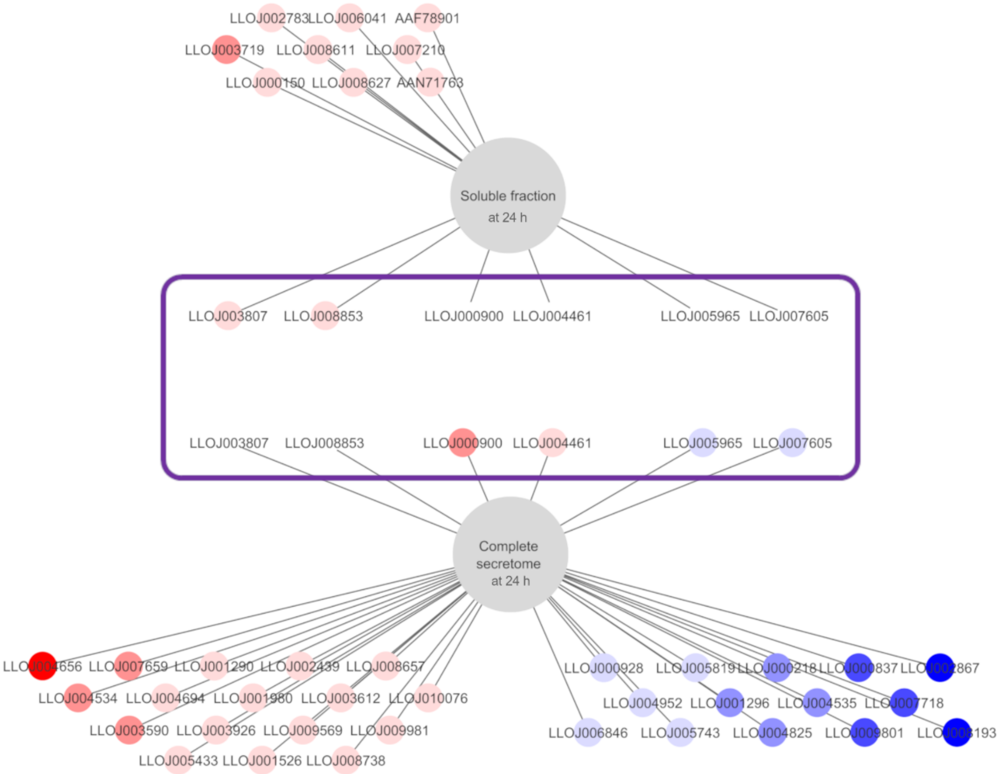


| VectorBase ID | Protein Name | Fold change at 24h |
|---|---|---|
| LLOJ004656 | Scramblase | 8.73 |
| LLOJ004534 | Prefoldin | 5.38 |
| LLOJ000900 | FKBP | 5.14 |
| LLOJ007659 | Coatomer delta subunit | 4.82 |
| LLOJ003590 | Programmed cell death protein 6 | 4.43 |
| LLOJ004694 | Adenylosuccinate lyase | 3.96 |
| LLOJ008738 | RNA binding protein 8A | 3.89 |
| LLOJ003926 | Kinesin | 3.63 |
| LLOJ001290 | Piruvate Kinase | 2.88 |
| LLOJ001980 | Carboxylesterase, beta esterase | 2.81 |
| LLOJ009569 | Protein canopy 4 | 2.73 |
| LLOJ002439 | Barrier-to-autointegration factor | 2.65 |
| LLOJ004461 | Glutathione S-transferase | 2.60 |
| LLOJ003612 | Proteasome subunit alpha 3 | 2.45 |
| LLOJ008657 | RNA exonuclease 1 | 2.32 |
| LLOJ009981 | Juvenile hormone-inducible protein | 2.12 |
| LLOJ001526 | Proteasome subunit alpha 2 | 2.11 |
| LLOJ010076 | unknow | 2.09 |
| LLOJ005433 | Phosphoglycerate mutase | 2.07 |

| VectorBase ID | Protein Name | Fold change at 24h |
|---|---|---|
| LLOJ005819 | Thioredoxin | -2.17 |
| LLOJ005743 | Disintegrin and metalloproteinase domain-containing protein 10 | -2.29 |
| LLOJ005965 | GTP-binding nuclear protein Ran | -2.33 |
| LLOJ007605 | Catalase | -2.48 |
| LLOJ004952 | Src substrate cortactin | -2.78 |
| LLOJ000928 | Bifunctional glutamate/proline-tRNA ligase | -2.89 |
| LLOJ006846 | 60S ribosomal protein S8 | -3.48 |
| LLOJ000218 | Vigilin | -4.26 |
| LLOJ001296 | SH3 domain-binding protein 5 | -4.41 |
| LLOJ004825 | Ribosomal protein S27 | -4.41 |
| LLOJ004535 | Eukaryotic translation initiation factor 3 | -4.50 |
| LLOJ009801 | Transketolase | -6.11 |
| LLOJ007718 | Acetyl-coenzyme A synthetase | -6.31 |
| LLOJ000837 | Tropomyosin | -7.65 |
| LLOJ002867 | Tyrosine-protein kinase-like otk | -9.07 |
| LLOJ008193 | Phosphoinositide-binding protein | -11.26 |

**Legend Figure A**: Differentially expressed proteins in LL5 cells 24h post-transfection with poly I:C are identified by VectorBase accession numbers. The upper network represents proteins identified in the soluble fraction (current study). The lower network represents proteins identified in the complete secretome (Martins-da-Silva et al. 2018). Proteins in the center (purple rectangle) were identified in both studies. Gradient color code corresponds to upregulated (red) to downregulated (blue) proteins (log fold change >|2|) in cells transfected with poly I:C compared to the mock-transfected control group. Proteins with log fold change in expression below |2| are not shown. Tables indicate VectorBase accession numbers with corresponding protein names and fold change values. Proteins highlighted in grey in the tables were identified in both studies.

h post-transfection(Martins-da-Silva et al. 2018)(Martins-da-Silva et al. 2018). But they did not have a marked expression change in the soluble fraction in the current study.

Figure B

| VectorBase ID | Protein Name | Fold change at 48h |
|---|---|---|
| LLOJ002783 | unknow | 5.02 |
| LLOJ000456 | CBM39 | 4.00 |
| LLOJ000150 | unknow | 3.85 |
| LLOJ007841 | CBM_14 | 3.12 |
| LLOJ007578 | Hemocytin | 2.93 |

| VectorBase ID | Protein Name | Fold change at 48h |
|---|---|---|
| LLOJ009307 | eIF2A | 2.87 |
| LLOJ003719 | Fasciclin | 2.83 |
| LLOJ008627 | unknow | 2.62 |
| LLOJ004575 | Peptidase_S8 | 2.49 |
| LLOJ006041 | Glyco_hydro_18 | 2.43 |
| LLOJ003528 | Peptidase_M10 | 2.27 |
| LLOJ008037 | Trypsin | 2.14 |
| AAF78901 | A_deaminase | 2.04 |
| LLOJ004461 | Glutathione S-transferase | -2.08 |
| LLOJ005377 | Phosphomannosemutase | -2.32 |

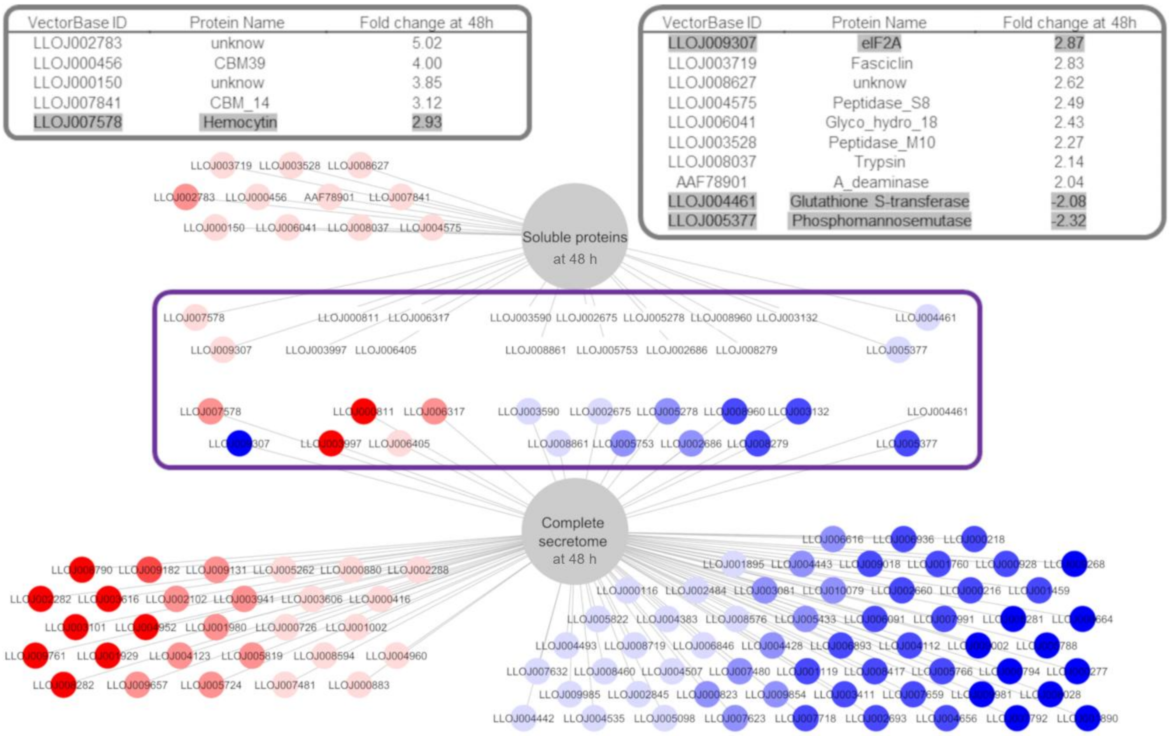


| VectorBase ID | Protein Name | Fold change at 48h |
|---|---|---|
| LLOJ008790 | Repressor splicing factor 1 | 10.29 |
| LLOJ001929 | Cytochrome B5 | 9.94 |
| LLOJ003101 | Heat shock 70 kDa protein cognate 5 | 9.62 |
| LLOJ004952 | Src substrate cortactin | 9.29 |
| LLOJ008282 | Actin-interacting protein 1 | 9.24 |
| LLOJ003997 | Coronin-6 | 9.02 |
| LLOJ009761 | 40S ribosomal protein S28-like | 9.02 |
| LLOJ003616 | Coracle | 8.76 |
| LLOJ002282 | Chordin-like protein 2 | 8.26 |
| LLOJ000811 | Eukaryotic translation initiation factor 3 | 8.15 |
| LLOJ009182 | Glutaredoxin | 7.96 |
| LLOJ001980 | Carboxylesterase beta esterase | 5.75 |
| LLOJ007578 | Hemocytin | 5.66 |
| LLOJ002102 | Glutamate carboxypeptidase | 5.40 |
| LLOJ006317 | Plasma glutamate carboxypeptidase | 5.25 |
| LLOJ003941 | Moesin/ezrin/radixin homolog 1 | 5.05 |
| LLOJ005819 | Thioredoxin | 4.95 |
| LLOJ009657 | 60S acidic ribosomal protein P2 | 4.94 |
| LLOJ004123 | 15 kDa selenoprotein | 4.37 |
| LLOJ005724 | Beta-1,3-galactosyltransferase 5 | 4.23 |
| LLOJ009131 | SH3 domain-binding glutamic acid-rich protein | 4.09 |
| LLOJ000880 | 14-3-3 protein zeta | 3.56 |
| LLOJ003606 | Glutamine--tRNA ligase | 3.53 |
| LLOJ001002 | unknow | 3.51 |
| LLOJ006405 | Laminin subunit beta-1 | 3.33 |
| LLOJ004960 | Zinc finger protein on ecdysone puffs | 3.24 |
| LLOJ007481 | Collagen alpha-2(IV) chain | 3.03 |
| LLOJ008594 | Superoxide dismutase | 2.99 |
| LLOJ000726 | Proliferation-assoc. prot. 2G4 | 2.66 |
| LLOJ002288 | Thioredoxin domain-containing protein 17 i | 2.39 |
| LLOJ000416 | Echinoderm microtubule-associated protein | 2.31 |
| LLOJ005262 | AP-2 complex subunit alpha | 2.17 |
| LLOJ000883 | Coactosin-like protein | 2.02 |

| VectorBase ID | Protein Name | Fold change at 48h |
|---|---|---|
| LLOJ002845 | 60S ribosomal protein L7 | -2.01 |
| LLOJ007632 | Beta-1 tubulin | -2.07 |
| LLOJ008460 | Peroxiredoxin 1 | -2.10 |
| LLOJ002484 | Clathrin heavy chain | -2.21 |
| LLOJ000116 | T-complex prot. 1 zeta | -2.26 |
| LLOJ002675 | Adenosylhomocysteinase | -2.31 |
| LLOJ008861 | T-complex prot. 1 beta | -2.42 |
| LLOJ005822 | 40S ribosomal prot. SA | -2.42 |
| LLOJ003590 | Programmed cell death protein 6 | -2.47 |
| LLOJ009985 | T-complex prot. 1 gamma | -2.52 |
| LLOJ004535 | Eukaryotic translation initiation factor 3 | -2.84 |
| LLOJ004507 | Synaptotagmin | -2.87 |
| LLOJ004493 | 40S ribosomal protein S3 | -2.93 |
| LLOJ001895 | 40S ribosomal protein S4 | -2.94 |
| LLOJ004383 | Vacuolar H[+]-ATPase 55kD | -3.06 |
| LLOJ004442 | Regulator of chromosome condensation | -3.15 |
| LLOJ005098 | Importin beta-3 | -3.23 |
| LLOJ008719 | Transaldolase | -3.45 |
| LLOJ006846 | 60S ribosomal protein S8 | -3.45 |
| LLOJ008576 | Ferritin heavy chain | -3.86 |
| LLOJ005278 | SPARC | -4.04 |
| LLOJ003081 | 40S ribosomal protein S13 | -4.14 |
| LLOJ005753 | Aspartate aminotransferase | -4.19 |
| LLOJ007480 | Collagen alpha-1(IV) chain | -4.22 |
| LLOJ006616 | Proteasome activator complex | -4.53 |
| LLOJ002686 | Laminin subunit gamma-1 | -4.70 |
| LLOJ004443 | Lipase 3 | -4.79 |
| LLOJ005433 | Phosphoglycerate mutase | -4.86 |
| LLOJ010079 | Leukotriene A-4 hydrolase | -4.90 |
| LLOJ000823 | Methionine aminopeptidase 2 | -5.52 |
| LLOJ007623 | Lecithin-cholesterol acyltransferase | -5.86 |
| LLOJ004428 | Protein NPC2 homolog | -5.97 |
| LLOJ009854 | GPRstn (toll-like) | -5.97 |
| LLOJ001459 | unknow | -6.06 |
| LLOJ003132 | Beta-mannosidase | -6.08 |
| LLOJ007991 | Farnesol dehydrogenase | -6.17 |
| LLOJ004656 | Scramblase | -6.19 |
| LLOJ008279 | Serine protease inhibitor | -6.23 |

| VectorBase ID | Protein Name | Fold change at 48h |
|---|---|---|
| LLOJ000218 | Vigilin | -6.29 |
| LLOJ008417 | BCR-associated protein | -6.41 |
| LLOJ000928 | Bifunctional glutamate/proline-tRNA ligase | -6.45 |
| LLOJ002660 | 60S ribosomal protein L27a | -6.59 |
| LLOJ000216 | Proteasome beta 2 | -6.63 |
| LLOJ007659 | Coatomer delta | -6.84 |
| LLOJ005377 | Phosphomannosemutase | -7.03 |
| LLOJ008960 | Apolipoprotein D | -7.24 |
| LLOJ006893 | 60S ribosomal prot. L23 | -7.36 |
| LLOJ005766 | 6-phosphogluconate dehydrogenase | -7.47 |
| LLOJ002693 | unknow | -7.57 |
| LLOJ001760 | Pom1 | -7.58 |
| LLOJ007718 | Acetyl-coenzyme A synthetase | -7.60 |
| LLOJ001119 | Transmembrane serine protease 9 | -7.64 |
| LLOJ004112 | 40S ribosomal prot. S5 | -7.82 |
| LLOJ003411 | Tubulin-specif. chaperone A | -7.84 |
| LLOJ006091 | Proliferating cell nuclear antigen | -7.93 |
| LLOJ009018 | Insulin-like growth factor-binding protein | -7.96 |
| LLOJ006936 | Vesicle amine transport prot | -7.99 |
| LLOJ008664 | NMDA receptor glutamate-binding chain | -8.05 |
| LLOJ009307 | Eukaryotic translation Initiation factor 3 B | -8.06 |
| LLOJ009981 | Juvenile hormone-inducible protein | -8.21 |
| LLOJ005788 | Valine--tRNA ligase | -8.28 |
| LLOJ000277 | Galectin | -8.29 |
| LLOJ005281 | Histone H2A.V | -8.30 |
| LLOJ001890 | Transcript. factor BTF3 | -8.63 |
| LLOJ009002 | Proteasome alpha 4 | -8.75 |
| LLOJ007792 | n-acetylgalactosaminyltransferase | -8.87 |
| LLOJ008028 | Chloride intracellular channel exc-4 | -8.92 |
| LLOJ009268 | RNA exonuclease 1 | -9.01 |
| LLOJ000794 | Histone H3 | -10.31 |

**Legend Figure B**: Differentially expressed proteins in LL5 cells 48h post-transfection with poly I:C are identified by VectorBase accession numbers. The upper network represents proteins identified in the soluble fraction (current study). The lower network represents proteins identified in the complete secretome (Martins-da-Silva et al. 2018). Proteins in the center (purple rectangle) were identified in both studies. Gradient color code corresponds to upregulated (red) to downregulated (blue) proteins (log fold change >|2|) in cells transfected with poly I:C compared to the mock-transfected control group. Proteins with log fold change in expression below |2| are not shown. Tables indicate VectorBase accession numbers with corresponding protein names and fold change values. Proteins highlighted in grey in the tables were identified in both studies.

## Discussion

This comparative analysis between the soluble and exosomal secreted proteomes of LL5 cells transfected with poly I:C reveals both overlapping and distinct features in the secretory response of these insect cells. Notably, although 48 proteins were identified as differentially regulated in both fractions across the 24 h and 48 h time points, their expression profiles often diverged depending on the secretory route. For instance, at 24 h post-transfection, proteins such as signal-induced proliferation-associated protein and prosaposin domain-containing protein were upregulated in the soluble fraction but not in exosomes, while other proteins like FKBP and GST_N were significantly modulated in exosomes only, suggesting differential sorting or retention mechanisms. By 48 h, although hemocytin and PMM showed concordant regulation across both compartments, eIF2A and GST_N displayed opposite or compartment-specific regulation (Basisty et al. 2020). These discrepancies underscore a dynamic and potentially compartmentalized cellular response to viral mimic stimulation.

These differences reflect the temporal and functional divergence of the secretory routes mobilized in response to viral mimicry. The soluble secretome may represent a more rapid, expansive means of extracellular signaling, incorporating metabolic enzymes, signaling ligands, and even components of translational machinery with potential immunomodulatory effects. In contrast, the exosomal pathway appears to orchestrate a more regulated and selective export of immune and stress-related proteins, possibly tuned for cell-to-cell delivery and long-range effects (Munoz-Perez et al. 2021). Together, the two secretomes illustrate distinct yet potentially complementary aspects of the innate antiviral landscape in sand fly cells.

Importantly, this study differs from Martins-da-Silva et al. (2018) not only in the focus on soluble secreted proteins but also in the cellular context captured. While exosomes represent a vesicle-based, potentially regulated route of intercellular communication, the soluble proteome likely reflects a range of secreted proteins, including freely diffusing mediators of local and systemic responses (Samuelson and Vidal-Puig 2018). The soluble fraction may also contain proteins related to acute-phase responses, stress signaling, or passive leakage, not captured within the exosomal compartment. This distinction highlights the complementary nature of both datasets: the exosomal proteome reveals targeted export of regulatory components, while the soluble proteome captures the immediate extracellular milieu, including potential effector proteins (Basisty et al. 2020). Together, the two studies provide a more integrated view of how LL5 cells modulate their secretory machinery in response to dsRNA analogs and may contribute differentially to cell-cell signaling, immune modulation, and antiviral defense.

**References**


Basisty, Nathan, Abhijit Kale, Ok Hee Jeon, et al. 2020. “A Proteomic Atlas of Senescence-Associated Secretomes for Aging Biomarker Development.” *PLOS Biology* 18 (1): e3000599. https://doi.org/10.1371/journal.pbio.3000599.

Martins-da-Silva, Andrea, Erich Loza Telleria, Michel Batista, Fabricio Klerynton Marchini, Yara Maria Traub-Csekö, and Antonio Jorge Tempone. 2018. “Identification of Secreted Proteins Involved in Nonspecific dsRNA-Mediated Lutzomyia Longipalpis LL5 Cell Antiviral Response.” *Viruses* 10 (1): 43–43. https://doi.org/10.3390/v10010043.

Munoz-Perez, Elena, Ainhoa Gonzalez-Pujana, Manoli Igartua, Edorta Santos-Vizcaino, and Rosa Maria Hernandez. 2021. “Mesenchymal Stromal Cell Secretome for the Treatment of Immune-Mediated Inflammatory Diseases: Latest Trends in Isolation, Content Optimization and Delivery Avenues.” *Pharmaceutics* 13 (11): 1802. https://doi.org/10.3390/pharmaceutics13111802.

Samuelson, Isabella, and Antonio J. Vidal-Puig. 2018. “Fed-EXosome: Extracellular Vesicles and Cell–Cell Communication in Metabolic Regulation.” *Essays in Biochemistry* 62 (2): 165–75. https://doi.org/10.1042/EBC20170087.

Shannon, Paul, Andrew Markiel, Owen Ozier, et al. 2003. “Cytoscape: A Software Environment for Integrated Models of Biomolecular Interaction Networks.” *Genome Research* 13 (11): 2498–504. https://doi.org/10.1101/gr.1239303.
